# A systematic review of randomized controlled trials of dietary interventions for weight loss in adults in the Middle East and north Africa region

**DOI:** 10.1111/cob.12434

**Published:** 2020-12-26

**Authors:** Hadeel Zaghloul, Hadya Elshakh, Abdullah Elzafarany, Odette Chagoury, Barbara McGowan, Shahrad Taheri

**Affiliations:** ^1^ Department of Medicine Weill Cornell Medicine Doha Qatar; ^2^ Department of Medicine Weill Cornell Medicine New York USA; ^3^ National Obesity Treatment Centre Qatar Metabolic Institute, Hamad Medical Corporation Doha Qatar; ^4^ Department of Diabetes King's College London London UK; ^5^ Department of Diabetes and Endocrinology Guy's and St Thomas' NHS Trust London UK

**Keywords:** diet, Middle East, north Africa, systematic review, weight loss

## Abstract

The prevalence and incidence of obesity, and associated complications, such as type 2 diabetes, in the Middle East and north Africa (MENA) region rank among the highest in the world. Little is known about the effectiveness of dietary weight loss interventions conducted in the MENA region. We conducted a systematic review of randomized clinical trials aiming to assess the effectiveness of dietary interventions for weight loss in the adult population originating from and residing in the MENA region. In accordance with PRISMA guidelines, PubMed, CINAHL, Cochrane, and EMBASE were systematically searched for randomized controlled trials (RCT) using dietary interventions for weight loss conducted in the MENA region. RCTs examining weight loss as an outcome in adults (≥ 18 years old) were included. The Cochrane Collaboration tool for assessing risk of bias was used to ascertain the quality of the eligible RCTs and the Template for Intervention Description and Replication for population health and policy interventions (TIDieR‐PHP) checklist was used to evaluate the reporting of the interventions. Twenty‐nine RCTs including 2792 adults from five countries in the MENA region met the search criteria. Study participants were predominantly middle‐aged females. Duration of follow up was mostly 3 months or less. Weight loss ranged from −0.7 to 16 kg across all intervention groups and the average weight loss was 4.8 kg. There was paucity of description of the weight loss interventions and variations amongst studies did not allow a meta‐analysis of findings. It was not possible to draw firm conclusions on the effectiveness of dietary weight loss interventions in the region. High quality studies using more structured interventions of longer duration with standardized outcome measures are needed in the MENA region to support clinical practice with evidence‐based interventions for obesity.

**Abbreviations**
ADA: American Diabetes Association
AHA: American Heart Association
BMI: body mass index
CHO: carbohydrate
CVD: cardiovascular disease
DASH: dietary approaches to stop hypertension
IDF: International Diabetes Federation
MENA: Middle East and north Africa
NCD: non‐communicable disease
PRISMA: Preferred Reporting Items for Systematic Reviews and Meta‐Analyses
RCT: randomized controlled trial
T2DM: type 2 diabetes mellitus

## 1 INTRODUCTION

Obesity is a significant global health problem affecting developed and developing countries alike. The prevalence of obesity over the past few decades has more than doubled resulting in about one third of the population having a body mass index (BMI) in the obese range.^1^ The rise in obesity prevalence has occurred across all age groups, geographical locations, and socioeconomic categories.^1^

The Middle East and north Africa (MENA) region is experiencing a significant challenge from population obesity and diabetes. A systematic review estimated that 25% to 82% of adults (with higher prevalence in women) and 7% to 45% of school children in the MENA region were within overweight or obese range.^2^ Another study compared the prevalence of obesity in 52 countries across eight different geographical locations, and found that women in the MENA region had the highest waist‐to‐hip ratio and the second highest BMI, after United States, compared to other regions.^3^ Similar findings were reported by another study that included 199 countries.^4^

Obesity is a key risk factor for non‐communicable diseases (NCD), such as cardiovascular disease (CVD),^5^ type 2 diabetes mellitus (T2DM),^6^ chronic kidney disease,^7^ several major cancers,^8^ musculoskeletal disease^9^ and mental health disorders.^10^ The rapid rise of obesity prevalence has led to a substantial increase in the prevalence of NCD in the MENA region, particularly at a younger age.^2^ The International Diabetes Federation (IDF) estimated that in 2017, 40 million people were living with diabetes in the MENA region and projected that this number will more than double to 86 million in 2045,^11^ placing the region as having the second highest prevalence of diabetes (9.2%). Beside the burden of disease and negative impact on quality of life, it has been estimated that NCDs are the leading cause of death in the MENA region accounting for about 60% of total deaths.^12^

Dietary interventions (with or without physical activity) are essential for the prevention and treatment of overweight and obesity in all populations. The effectiveness of dietary interventions in populations in the MENA region, however, has not been systematically reviewed and there are few clinical guidelines for the prevention and management of obesity in MENA countries. We conducted a systemic review of dietary interventions for weight loss in the MENA region. We included studies carried out in adults (≥ 18 years old) with comparators (control or other intervention) that reported on weight loss as an outcome. We aimed to identify key interventions (and assess their effectiveness) that may inform guidelines for prevention and treatment of obesity in the region. Furthermore, our systematic review aimed to identify research gaps for tackling obesity in a region with one of the highest prevalence of obesity.

## 2 METHODS

### 2.1 Protocol and Registration

This systematic review was conducted according to the PRISMA guidelines,^13^ and used a pre‐defined protocol registered with PROSPERO (CRD42017068811). The full systematic review described in the protocol evaluates all randomized controlled trials (RCTs) of dietary interventions conducted in the MENA region. For the purposes of this report, the focus was on interventions for adults. Furthermore, the current report does not address the use of dietary supplements. For a more comprehensive review, studies that reported changes in weight as an outcome measure were included.

### 2.2 Eligibility criteria

#### 2.2.1 Participants

All studies carried out in adults (≥ 18 years old) were included. Participants had to originate from the MENA region, which was defined to include the following countries: Algeria, Bahrain, Cyprus, Egypt, Iran, Iraq, Israel, Jordan, Kuwait, Lebanon, Morocco, Oman, Palestine, Qatar, Saudi Arabia, Somalia, Sudan, Syria, Tunisia, Turkey, United Arab Emirates and Yemen.

#### 2.2.2 Interventions

All studies using dietary interventions with the aim of, or reporting, weight loss as a key outcome were included. Any intervention that used any medicinal products, surgical interventions, or nutritional supplements for weight loss were excluded. No filter on the duration of intervention was placed.

#### 2.2.3 Types of comparators

Included studies all had comparator groups. This included comparison with no intervention or comparison between various intervention modalities.

#### 2.2.4 Type of outcome measures

Included studies had to report on weight or weight loss (measured in kilograms or change in BMI) as an outcome. This had to be measured at baseline and then at least one time point from baseline. Outcomes reflecting glycaemic control were of secondary interest in this review and are included if reported in publications.

#### 2.2.5 Types of studies

Only RCTs were considered for inclusion.

#### 2.2.6 Study selection

The inclusion and exclusion criteria are listed in Table 1. Broad inclusion criteria were used for eligibility assessment of titles and abstracts, which was performed by two reviewers independently (Hadeel Zaghloul, Hadya Elshakh). Conflicts were resolved by consensus. Full texts of potentially relevant studies were obtained and assessed against the inclusion/exclusion criteria independently by two reviewers (Hadeel Zaghloul, Abdullah Elzafarany). Conflicts were resolved by consensus or by consultation of a third party (Shahrad Taheri).

**TABLE 1:** Inclusion and exclusion criteria

Inclusion criteria	Exclusion criteria
1. Human Research 2. Adults > = 18 years 3. MENA region 4. Dietary Intervention 5. Weight as an outcome 6. Randomized Controlled Trials 7. Studies of any duration 8. All languages	1. Animal Studies 2. Bariatric surgery/surgical weight loss 3. Medicinal interventions 4. Nutritional supplements

#### 2.2.7 Information sources

The search strategy and terms were developed by the research team. Studies were identified by searching the following electronic databases: PubMed, Medline, CINAHL, Cochrane, and EMBASE. Databases were searched from inception to February 2020. Search terms (keywords, subject headings, and so on) applicable to the subject areas of “diet” and “Middle East” and “north Africa”, as defined in this systematic review, were used and also harvested from within the content of the databases listed above. The search terms were reviewed by several authors and are provided in additional file on request; both subject headings and keywords were used in search string construction. Boolean Operators and truncation were inserted into searches at all points in which these functions were seen as an appropriate enhancement to a search. The search string used is available in the supplementary material.

No filters for language or years of publication were applied to the searches. Searches within the grey literature were conducted in order to harvest relevant works that might not be uncovered through only searching through the contents of traditional scholarly databases. Finally, a manual search was performed by two independent reviewers to retrieve any articles that were not identified in the initial search. The reference lists of relevant articles (articles that met the inclusion criteria) were checked to ensure that all relevant articles were identified.

#### 2.2.8 Study quality and risk of bias assessment

The Cochrane Collaboration tool for assessing risk of bias was used to ascertain the quality of the eligible RCTs. Cochrane Collaboration tool assesses RCT validity based on five domains (selection, performance, attrition, reporting, and other). The Template for Intervention Description and Replication for population health and policy interventions (TIDieR‐PHP) checklist^14^ was used to evaluate the quality of the description of the interventions in the publications included in the review.

#### 2.2.9 Data extraction

A data extraction form was developed and piloted for the first five articles and then adjusted accordingly. Data were extracted by one author (Hadeel Zaghloul) and verified by another (Abdullah Elzafarany). Discrepancies were resolved by consensus or by consultation of a third party (Shahrad Taheri). We extracted study identification details, study design and methods, population characteristics, inclusion and exclusion criteria, interventions and outcomes.

#### 2.2.10 Analysis

No statistical analysis or meta‐analyses were possible due to the extent, diversity and quality of data available. Data from the eligible trials was extracted and reported in a systematic manner.

## 3 RESULTS

### 3.1 Study selection

**FIGURE 1:**
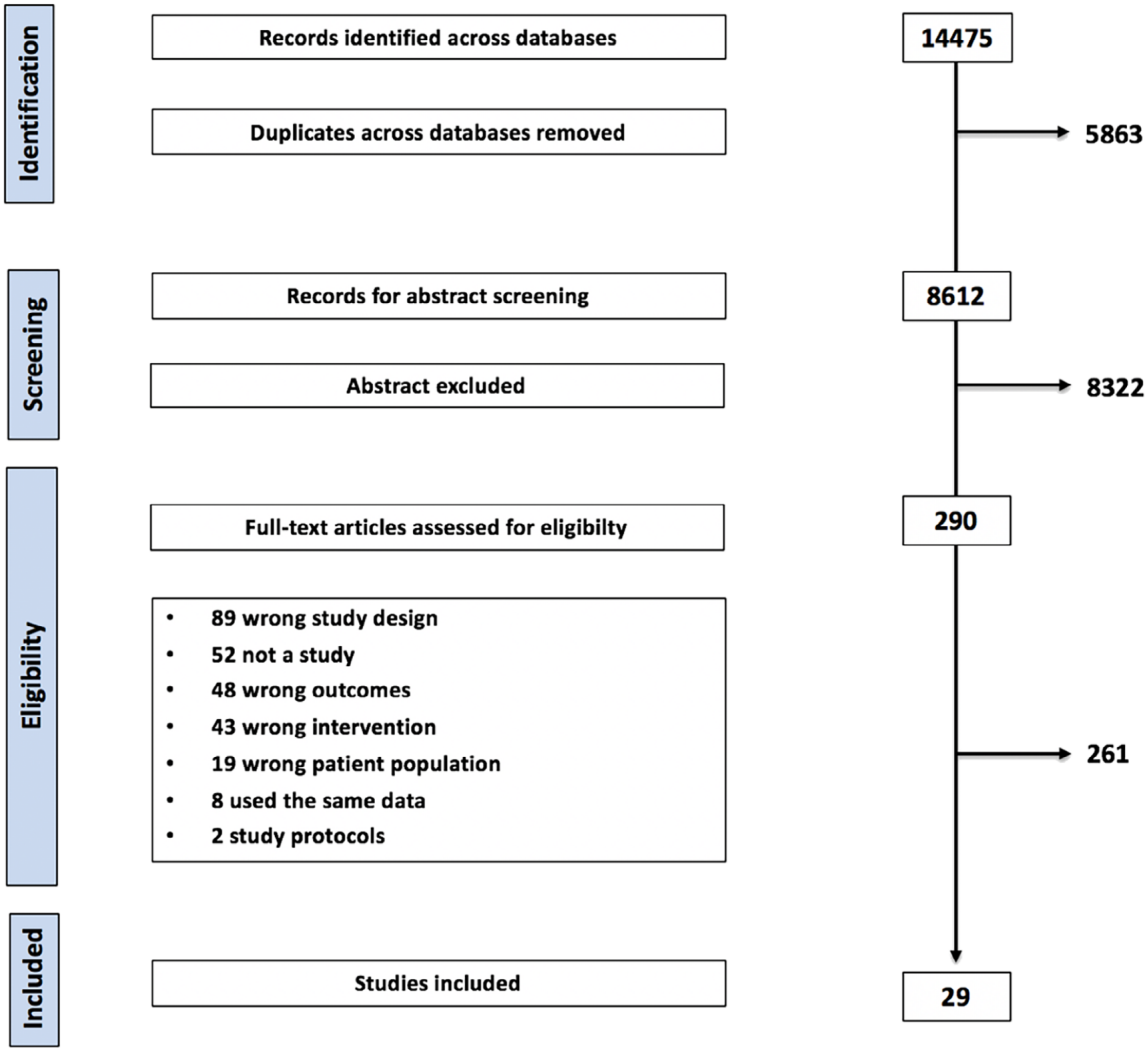
Flowchart summarizing study selection

The electronic database search identified 8612 potentially relevant articles for screening. No additional results were identified through manual searches. Following title and abstract screening, 290 articles remained. After examining the full text in more detail, 29 RCTs were deemed eligible for inclusion in the review. Interventions that used dietary intervention in combination with physical activity (three studies) or behavioural modification (four studies) were also included. Figure 1 shows the study selection process.

### 3.2 Study characteristics

Table 2 provides the details of the 29 included studies. A total of 2792 adults were recruited in the 29 RCTs. All but one trial^15^ recruited people with overweight and obesity. Sixteen trials (55%) included women only and four (14%) included only post‐menopausal women. Seven trials (24%) recruited individuals with the metabolic syndrome, seven (24%) with T2DM, 2 (7%) reported that subjects had diabetes but did not specify which type, and one trial (3%) recruited individuals with psoriasis. Ten trials (35%) recruited individuals with no reported obesity complications or comorbidities. One trial (3%) did not report on the presence or absence of obesity complications.^16^

**TABLE 2:** Baseline characteristics of studies included in the systematic review

Trial (Author, years)	Country	Recruited N (Total)	Drop‐out (%)	Follow up (months)	Obesity complications/Comorbidity	Mean age years (SD)	Female (%)	Mean BMI kg/m2 (SD)	Weight Loss intervention	Comparator	Outcome measures
Abd El‐Kader et al., 2016^44^	Saudi Arabia	103 Data reported n = 80	12.6%	3	None	52.64 (6.13)	100%, post‐menopausal	Diet: 33.71 (3.12) Control: 34.11 (3.54)	Group 1 – Balance d low‐calorie diet (LED) (1200 kcal/day) + aerobic exercise training program	Control	BMI, TNF‐a, IL‐6, CRP, ICAM‐1, VCAM‐1, PAI‐1: Ac
Abedi et al., 2010^16^	Iran	76 Data reported n = 64	15.8%	6	NR	Diet: 51.4 (4.9) Control: 51.6 (5.7)	100%, post‐menopausal	Diet: 30.1 (6.2) Control: 30.8 (30.8)^a^	Group 1 ‐ Educational sessions: five servings of fruit and vegetables, whole grain foods, high fibre foods, fish (two times per week), saturated fat <10% of energy, cholesterol <300 mg/day, and salt <5 g/day, consumption of trans‐fatty acids as low as possible	Control	Wt, BMI, WHR, SBP, DBP, TC, TG, LDL, HDL, FBG, dietary intake
Al‐Mutairi et al., 2014^45^	Kuwait	262 Data reported n = 262	NR	6	Psoriasis	46.9 (6.4)	64.5%	Diet: 29.3 (4.2) Control: 29.5 (5.2)	Group 1: LED (≤1000 kcal/day) calculated according to the resting energy outflow	Control	Wt, WC reduction, PASI 75(%), BSA, TG, TC
Al‐Sarraj et al., 2009^17^	United Arab Emirates	56 Data reported n = 39	30.4%	3	Metabolic Syndrome	NR (18‐50 years)	64.1%	Group 1: 38.7 (7.6) Group 2: 33.5 (6)	Group 1: CHO restricted diet: (20‐25% CHO) for 12 weeks Group 2:6‐weeks CHO restricted diet followed by a 6‐weeks conventional low‐fat diet as prescribed by the AHA^b^		Wt, BMI, WC, Body fat, Trunk fat, SBP, DBP, TC, LDL, HDL, TG, FBG, Insulin, HOMA, MS, dietary intake, inflammatory markers
Azadbakht et al, 2005^24^	Iran	116 Data reported n = 116	0%	6	Metabolic Syndrome	41.2 (12.3)	70%	Group 1:29.9 (10.1) Group 2:29.8 (10.3) Control: 29.5 (9.9)	Group 1: Wt reducing diet; 500 kcal deficit from needs based on body Wt with macronutrient composition similar to control diet. Group 2: DASH^c^ diet; 500 kcal deficit from needs based on body Wt	Control (observational not randomized)	WC, Wt, HDL, TG, SBP, DBP, FBG
Azadbakht et al, 2007^23^	Iran	42 Data reported n = 42	0%	2	Metabolic Syndrome	NR	100%, post‐menopausal	NR	Group 1: DASH^c^ diet with soy‐nut Group 2: DASH^c^ diet with soy‐protein Group 3: DASH^c^ diet with red meat All received all three diets (crossover design) and had two washout periods (each washout for 4 weeks)		Wt, WC, SBP, DBP, FBG, TG, HDL, LDL, TC, insulin, HOMA‐IR, C‐peptide, Apolipoprotein AI, Apolipoprotein B100, dietary intake
Darvish et al, 2012^46^	Iran	50 Data reported n = 43	14%	2	T2DM (1 year from diagnosis by OGTT)	Group 1: 51.7 (7.9) Control: 56 (5.7)	68% (reported as 79.1%)	Group 1:28.6 (5.8) Control: 28.6 (3)	Group 1: Cashew nuts 10% of diet	Control	Wt, BMI, WC, TC, TG, HDL, LDL, FBG, Insulin
Delvarianzadeh et al., 2006^25^	Iran	144 Data reported n = 43	NR	1	T2DM	52.1 (10)	NR	NR	Group 1:50% to 60% of caloric intake from CHO, 10% to 20% protein, <30% fat.	Control	Wt, SBP, DBP, FBG, TC, TG
Elhayany et al., 2010	Israel	259 Data reported n = 179	31%	12	T2DM	Group 1:55.5 (6.5) Group 2: 57.4 (6.1) Group 3: 56 (6.1)	48%	Group 1: 31.4 (2.8) Group 2: 31.1 (2.8) Group 3: 31.8 (3.3)	Group 1: low CHO Mediterranean (LCM)^d^ Group 2: Traditional Mediterranean^d^ (TM), Group 3: ADA^e^ diet		Wt, BMI, WC, FBG, HbA1c, TC, LDL, HDL, TG, insulin, HOMA
Esmaeili et al., 2014^47^	Iran	75 Data reported n = 60	20%	2	Metabolic Syndrome	45.41 (10.87)	85.2%	32.99 (5.01)	Group 1: Education on Razavi Style diet^f^	Control	Wt, WC, SBP, DBP, TC, HDL, LDL, TG, FBG, Insulin, BMI, Metabolic Syndrome severity
Fathi et al, 2016^48^	Iran	75 Data reported n = 75	22.7%	2	None	Group 1: 34.8 CI (32.8, 36.8) Group 2:35.2 CI (33, 37.3) Control: 36.5 CI (34.4, 38.7)	100%, pre‐menopausal	Group 1: 28.8 CI (27.9, 29.6) Group 2:29.5 CI (28.7, 30.3) Control: 28.9 CI (28.1, 29.7)	Group 1: weight maintenance diet, containing two additional servings/day (a total of four servings/day) of dairy products from low‐fat milk Group 2: weight maintenance diet, containing two additional servings/day (a commercial kefir drink	Control	Wt, BMI, WC, dietary intake
Hariri et al., 2014^15^	Iran	48 Data reported n = 40	16.7%	2	T2DM	Group 1:56.9 (1.81) Control: 53.6 (1.6)	52.5%	Group 1:26.68 (0.71) Control: 26.58 (0.73)	Group 1:200 mL probiotic soymilk per day	Control: conventional soymilk	Wt, BMI, WHR, SBP, DBP, dietary intake
Hosseinpour‐Niazi, 2015^49^	Iran	40 Data reported n = 31	22.5%	5	T2DM	58.1 (6)	77.4%	Group 1:27.7 (0.6) Group 2:27.8 (0.6)	Group 1: legume‐based TLC diet^g^ Group 2: legume‐free TLC diet^g^ All received both diets (crossover design). Each diet for 8 weeks, with a washout for 4 weeks in‐between.		BMI, WC, SBP, DBP, FBG, fasting insulin, TG, HDL, LDL, TC, dietary intake
Jahangiry et al., 2015^50^	Iran	160 Data reported n = 160	26.9%	6	Metabolic Syndrome	44.2 (10)	33.7%	30.1 (4.6)	Calorie‐restricted tailored diet	Control	MS status, Wt, BMI, elevated SBP, elevated DBP, impaired FBG, elevated TG, low HDL, Abdominal obesity %, physical activity, nutritional status, self‐reported health status
Kalter‐Leibovici et al., 2010^19^	Israel	201 Data reported n = 201	10.4%	12	1 or more components of Metabolic Syndrome	Group 1: 43.8 (5.6) Group 2: 44 (5.9)	100%	Group 1:34 (3.1) Group 2:33.8 (2.8)	Group 1: caloric reduction with intensive intervention; 11 individual and 11 group counselling sessions with a dietitian and 22 physical activity group sessions per year. Group 2: caloric reduction with moderate intervention; three individual and two group sessions with dietician		Metabolic Syndrome Component, WC, TG, HDL, FBG, SBP, DBP, Wt, HOMA‐IR, hs‐CRP, 2hppG, physical activity, QOL
Madjd et al., 2015^51^	Iran	71 Data reported n = 62	12.7%	6	None	Group 1: 31.7 (6.8) Group 2: 32.2 (6.9)	100%	Group 1:33.5 (3.6) Group 2:33.9 (3)	All got LED (high CHO and low saturated fat) and advice to gradually increase activity levels to achieve 60 minutes of moderate activity 5 days/wk with Group 1: Diet beverage (250 mL) after main meal Group 2: water (250 mL) after main meal		Wt, BMI, WC, TC, HDL, LDL, TG, FBG, 2hpp HbA1c, Insulin, HOMA‐IR, dietary intake
Madjd et al., 2016^52^	Iran	89 Data reported n = 89	9%	3	None	Group 1: 31.78 (6.81) Group 2: 32.20 (6.94)	100%	Group 1: 32.05 (3.94) Group 2: 32.14 (3.20)	Group 1: standard low‐fat yogurt + LED Group 2: probiotic yogurt + LED		Wt, BMI, WC, TC, HDL, LDL, TG, FBG, 2hPPG, Hba1c, fasting insulin, HOMA
Mahdavi et al., 2016^53^	Iran	90 Data reported n = 49	45.6%	6	None	Group 1: 28.40 (7.98) Group 2:27.02 (6.37)	100%	Group 1: 32.28 (2.90) Group 2: 33.22 (3.16)	Group 1: Balanced LED Group 2: Balanced LED+ nutrition education		Wt, BMI, processes of change
Mohammad‐Shahi et al., 2015^54^	Iran	60 Data reported n = 60	NR	3	None	34.15 (5.34)	100%	Group 1:34.9 (3.9) Group2 (Control): 34.7 (5.07)	Group 1: nutritional education	Control	Wt, physical activity levels, BMI, WC, HC, WHR, Body fat % TNF‐a, hs‐CRP
Nourieh et al., 2012^22^	Iran	30 Data reported n = 24	20%	2.5	None	37.7 (1.3)	100%, non‐menopausal	30.85 (0.83)	Group 1:4‐weeks soymilk period Group 2:4‐weeks cow's milk Both diets: 50‐60% CHO, 15‐20% protein, and < 30% total fat. All received both diets (crossover design). Each diet for 4 weeks, with a washout for 2 weeks in‐between.		Wt, TG, TC, HDL, LDL, hs‐CRP, IL‐6, dietary intake, physical activity
Parham et al., 2014^55^	Iran	48 Data reported n = 44	8.3%	8	T2DM	Group 1: 53 (10) Control: 50 (11)	75%	Group 1: 32.16 (6.58) Control: 30.24 (4.03)	Group 1:2 snacks of 25 g pistachios per day for 12 weeks + morning and evening servings (A) All received both diets (crossover design). Each diet for 12 weeks, with a washout for 8 weeks in‐between.	Control (B): no nuts	BMI, SBP, DBP, FBG, HbA1c, HOMA‐IR, CRP
Pourahmadi et al., 2015^21^	Iran	80 Data reported n = 75	6.3%	20 days	None	NR (20–30 years)	100%	Group 1:28.22 (0.35) Control: 28.28 (0.29)	Group 1: Tomato juice 2×/day	Control: water	Wt, BMI, dietary intake, antioxidant status
Rahimian et al., 2010^56^	Iran	21 Data reported n = 21	NR	1.5	Hypertension	NR (30‐49 years)	100%	33.54	Group 1: aerobic training+ LED Group 2: LED only group		Wt, BMI, SBP, DBP, WC, WHR, TC, TG, HDL, LDL, FBG, Insulin, Insulin Resistance, Renin, Aldosterone
Rajaie et al., 2012^57^	Iran	39 Data reported n = 30	23.1%	3.5	Metabolic Syndrome	42.4	100%	33	Group 1: high‐CHO (60% to 65% CHO), 20% to 25% fats Group 2: moderately‐restricted CHO (43% to 47% CHO), 36% to 40% fats diet All received both diets (crossover design). Each diet for 6 weeks, with a washout for 2 weeks in‐between.		Wt, BMI, fat mass, fat %, Lean body mass, WC, HC, WHR, FBG
Ramezankhani et al., 2015^58^	Iran	40 Data reported n = 40	0%	4	None	40.5 (5.05)	100%	Group 1:31.3 (2.44) Group 2:31.5 (2.47) Group 3:31.32 (2.51) Control: 31.95 (2.44)	Group 1: aerobic exercise Group 2: LED Group 3: aerobic exercise+LED	Control	Wt, BMI, WHR, preptin, FBG, HOMA‐IR
Salar et al., 2016^59^	Iran	75 Data reported n = 72	4%	2	T2DM	Group 1:52.18 (2.43) Group 2:50.19 (7.08) Control: 51.97 (6.42)	100%, post‐menopausal	Group 1:29.81 (2.81) Group 2:29.37 (2.44) Control: 30.54 (2.68)	Group 1: balanced diet+30 g/day canola oil Group 2: balanced diet+30 g/day rice bran oil	Control: balanced diet+30 g/ day sunflower oil	Wt changes, TC, TG, LDL‐c, HDL, Non‐HDL, dietary intake
Shai et al., 2008^27^	Israel	322 Data reported n = 322	15.5%	24	DM (n = 46), CHD (n = 118)	52 (7)	14%	30.9 (3.6)	Group 1: low‐fat, LED was based on AHA^b^ guidelines. Group 2: moderate‐fat, LED, Mediterranean^d^ diet Group 3: low‐CHO, non‐LED diet		Wt, WC, SBP, DBP, HDL, TG, LDL, TC/HDL, CRP, Adiponectin, Leptin, FBG, Insulin, HOMA‐IR, dietary intake, energy expenditure, urinary ketones
Tabesh et al., 2012^26^	Iran	60 Data reported n = 60	NR	2	DM	NR (30‐60 years)	100%	Group 1: 29.25 (0.88) Group 2: 29.83 (0.68) Group 3: 29.2 (1.26)	Group 1: low energy dense: (CHO: 65%, fat 25% of energy). (LD) Group 2: high energy dense (CHO: 55% and fat: 35%), (HD)	Group 3: normal‐energy‐dense diets (CHO: 60% and fat: 30%). (ND)	Wt, BMI, WC, FBG, HbA1c, TC, LDL, HDL, TG, insulin, HOMA‐IR
Razavi Zade et al., 2016^60^	Iran	60 Data reported n = 60	10%	2	NAFLD	Group 1:39.7 (7.3) Group 2:42.8 (10.6)	50%	Group 1:28.5 (3.2) Group 2:28.3 (3.3)	All received a LED with 52‐55% CHO, 16‐18% protein, 30% fats Group 1: DASH diet; 52‐55% CHO, 16‐18% protein, 30% fats Group 2: Control diet		Grade of fatty liver, WC, HC, AST, ALT, FBG, Insulin, HOMA‐IR, HOMA‐B, QUICKI, TG, VLDL‐C, TC, LDL, HDL, TAC, GSH, MDA

Abbreviations: ALT, alanine aminotransferase; APA, Apoprotein A; APB, Apoprotein B; AST, aspartate aminotransferase; BMI, body mass index; BSA, body surface area; CHD, coronary heart disease; CRP C‐reactive protein; DBP, diastolic blood pressure; DM, diabetes mellitus; FBG, fasting blood glucose; GSH, total glutathione; HC, hip circumference; HDL, High density lipoprotein cholesterol; HOMA‐B, homeostatic model assessment beta cell function; HOMA‐IR, homeostatic model assessment of insulin resistance; hs‐CRP, high sensitivity C‐reactive protein; IL‐6, Interleukin‐6; ICAM‐1, inter‐cellular adhesion molecule; LDL, low density lipoprotein; MDA, malondialdehyde; NAFDL, non‐alcoholic fatty liver disease; NR, not reported; OGTT, oral glucose tolerance test; PAI‐1, plasminogen activator inhibitor‐1 activity; PASI, psoriasis area and severity index; QOL, quality of life; SBP, systolic blood pressure; TAC, total antioxidant capacity; TC, total cholesterol; TG, triglycerides; TNF‐a, tumour necrosis factor alpha; VCAM‐1, vascular cell adhesion molecule; VLDL, very low density lipoprotein‐cholesterol; WC, waist circumference; Wt, weight; WHR, waist to hip ratio; QUICKI, quantitative insulin sensitivity check index; 2hPPG, 2 hour post prandial glucose.

^a^ Most likely error in SD reporting. Author contacted for verification; not verified.

^b^ AHA (American Heart Association) diet: The AHA recommends a diet that will reduce risk of CVD. It recommends that individuals consume a variety of fruits, vegetables, and grain products, especially whole grains; choose fat‐free and low‐fat dairy products, legumes, poultry, and lean meats; and eat fish, preferably oily fish, at least twice a week.

^c^ DASH (Dietary Approaches to Stop Hypertension) diet: DASH diet is especially recommended for people with hypertension or prehypertension to help control blood pressure. In addition to being a low sodium plan, the DASH diet is based on eating foods rich in fruits and vegetables, and low‐fat or non‐fat dairy, with whole grains. It is a high fibre, low to moderate fat diet, rich in potassium, calcium, and magnesium.

^d^ Mediterranean diet: A Mediterranean diet is based on traditional healthy eating habits of people from countries bordering the Mediterranean Sea. It is high in vegetables, fruits, legumes, nuts, beans, cereals, grains, fish, and unsaturated fats such as olive oil. Usually, it also has a low intake of meat and dairy foods.

^e^ ADA (American Diabetes Association): ADA diet is recommended by the ADA for diabetics. It involves meal planning such as carb counting and glycaemic index.

^f^ Razavi style diet: Dietary pattern driven from the text known as “Resaleh Zahabieh” meaning “Golden Letter” and belongs to Ali Ibn Musa (Imam Reza), the eighth Imam of Shiite sect of Islam. It recommends appropriate quantity, quality, feeding times and a suitable diet for each season and each month.

^g^ TLC: Therapeutic lifestyle changes (TLC) diet aims to reduce CVD risk. It recommends that 25‐30% of caloric intake is from fat, mainly unsaturated fat.

Twenty‐three trials (79%) were conducted in Iran, three (10%) in Israel, one (3%) in Saudi Arabia, one (3%) in Kuwait and one (3%) in the United Arab Emirates. Only one study (3%)^17^ recruited participants with a mean BMI ≥35 kg/m^2^ in only one arm (the mean BMI in the other arm was <35 kg/m^2^). Fourteen trials (48%) recruited participants with mean BMI ≥30 kg/m^2^. Two trials (7%) did not report on BMI.

Seventeen trials (59%) recruited middle‐aged individuals and only four trials (14%) recruited individuals with a mean age < 35 years. Two trials (7%) did not report the mean age of individuals. One trial (3%) followed up the intervention for 2 years,^18^ two trials (7%) for 1 year,^19^, ^20^ one trial (3%) for 8 months, four trials (14%) for 6 months and the majority of trials followed up on the intervention for less than 6 months. The shortest follow up duration was 20 days.^21^ Fourteen (48%) of the trials used a control group that received no intervention and 18 studies (62%) compared between different interventions.

**FIGURE 2:**
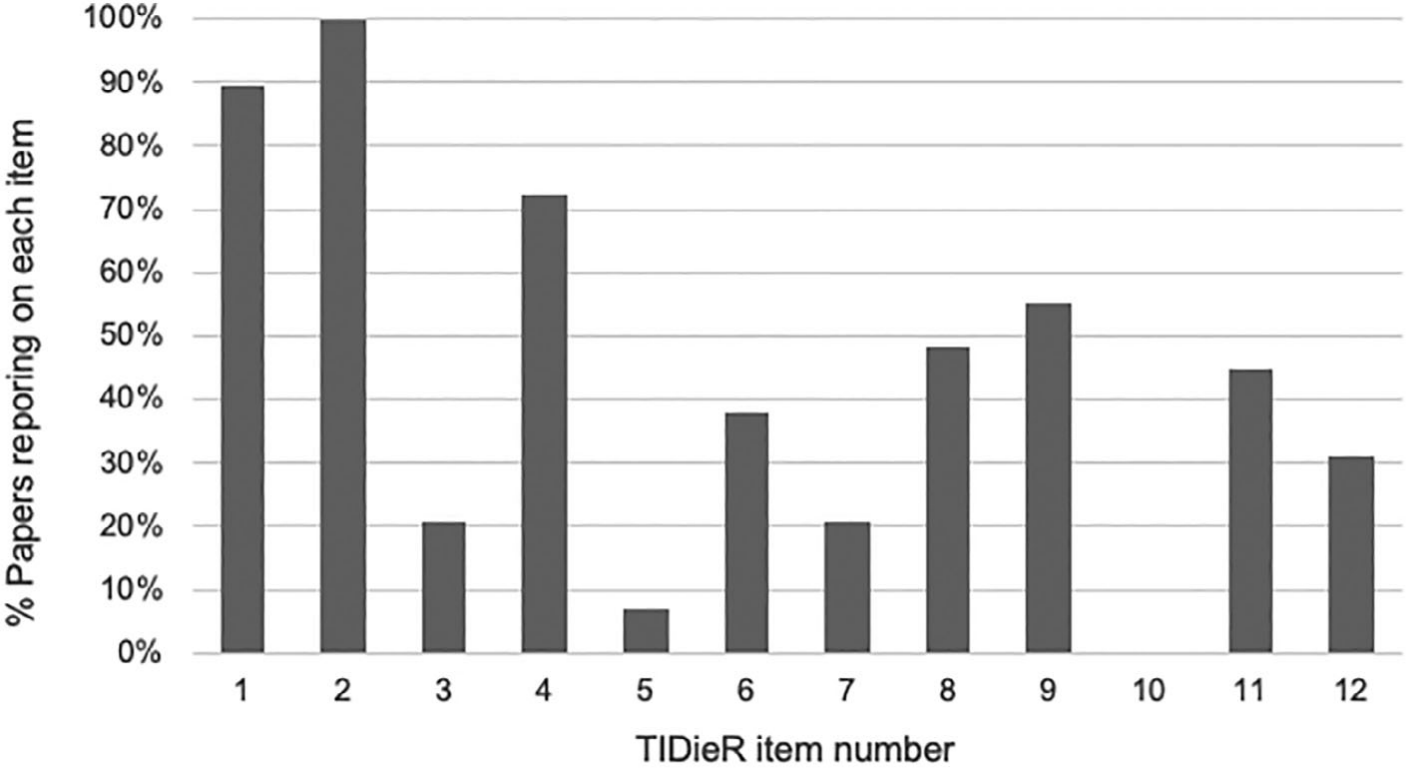
Percentage of studies reporting on TIDieR checklist items: 1 = Name or phrase describing intervention; 2 = Rationale, theory, goal of intervention; 3 = Materials used and where to access; 4 = Procedures; 5 = Expertise, background and training of each intervention provider; 6 = Mode of delivery; 7 = Type of location where intervention occurred; 8 = Number of times intervention delivered; what time period; no. sessions; schedule and duration/intensity/dose; 9 = If intervention personalized/titrated/adapted and how/when; 10 = If intervention was modified during study and changes described; 11 = If intervention adherence or fidelity was assessed; how and by whom (PLANNED); 12 = If intervention adherence or fidelity was assessed; describe extent delivered as planned (ACTUAL)

Figure 2 and supplenentary Table 1 provide a summary TIDieR‐PHP checklist for all the included studies. The majority of studies (79%) did not adequately describe the materials used in the intervention. Approximately half the studies did not report if the interventions were tailored to individuals and none reported any modifications to the intervention after study initiation. Most studies did not detail where the interventions were delivered (79%), the mode of delivery (62%) nor the duration and frequency of sessions (52%). Few studies (7%) described the intervention provider expertise and any relevant training they received for intervention delivery.

Twelve trials (41%) used energy restricted diets, six (21%) used fat restriction, and three trials (10%) used low carbohydrate (CHO) diets in at least one arm of their interventions. Three trials (10%) described the use of Dietary Approaches to Stop Hypertension (DASH) diet, two trials (7%) described using a Mediterranean diet and one trial (3.%) described using AHA (American Heart Association) diet. Two trials (7%) investigated the effect of supplementing diets with nuts, one using cashews (10% of caloric intake) and the other using pistachios (two snacks of 25 g). Three trials (10%) described the use of dairy products, two (7%) used soymilk, one (3%) used tomato juice and one (3%) used diet beverages and water. Only three trials (10%) reported on providing exercise as a component of the intervention in at least one arm.

### 3.3 Outcomes

A detailed description of the weight loss outcomes of the interventions is provided in Table 3. Twenty‐four studies reported change in weight (kg), and 20 studies reported a change in BMI kg/m^2^. One study reported only a percentage change in weight.^22^ Weight loss ranged from −0.7^23^ to 16 kg^24^ across all intervention groups and from −1.6^25^ to 3.44 kg^26^ across all control groups. The average weight loss was 4.81 kg in intervention groups and 0.15 kg in control groups. The change in BMI ranged from −0.1 to 4.68 kg/m^2^ in the intervention groups and from −0.28 to 0.4 kg/m^2^ in the control groups. It is important to highlight some of the key positive findings in the better‐quality studies that were performed in the MENA region. Shai et al. carried out a 2‐year trial, where 322 people (Table 2) were randomly assigned to one of three diets: low‐fat (energy intake limited to energy intake of 1500 kcal per day for women and 1800 kcal per day for men); Mediterranean, energy restricted; or low‐CHO, energy unrestricted.^27^ The rate of adherence to the study diet was reported to be 95.4% at 1 year and 84.6% at 2 years. Mean weight loss in the whole group of 322 participants was 2.9 kg (SD 4.2), 4.4 kg (SD 6) and 4.7 kg (SD 6.5) for the low fat, Mediterranean and low CHO diets, respectively. Among the 272 participants who completed the intervention, mean weight loss was 3.3 kg (SD 4.1), 4.6 kg (SD 6), and 5.5 kg (SD 7), respectively for the diets. The study found favourable effects on lipids with the low CHO diet and on blood glucose control with Mediterranean diet. The study was conducted at a workplace, which explains the high percentage of men (86%) in the study, making it less generalizable. Only 14% of the population had T2DM, and diabetes remission was not reported, although the greatest and most sustained reduction in fasting glucose and insulin amongst people with diabetes occurred with the Mediterranean diet. Azadbakht et al. studied the impact of diet on individuals (71% women) with the metabolic syndrome. They compared the DASH diet (reduced energy by 500 kcal, increased consumption of fruits, vegetables and low‐fat dairy) to a control diet and a 500 kcal energy restricted diet promoting healthy food choices.^24^ Subjects (N = 116) were followed up for 6 months. The DASH diet was found to be superior in weight loss and improving metabolic abnormalities. However, several discrepancies in reporting of findings have been highlighted including an unexpectedly large weight loss.^28^

**TABLE 3:** Weight loss outcomes of included studies

Trial (Author, year)	Weight kg (SD) (before)		Weight loss kg (SD)		Weight kg (SD) (after)		*P* value	BMI kg/m^2^ (SD) (before)		BMI kg/m^2^ (SD) (after)		*P* value
	Diet	Control	Diet	Control	Diet	Control		Diet	Control	Diet	Control	
Abd El‐Kader et al, 2016^44^	90.82 (6.84)	91.13 (6.51)	NR	NR	NR	NR	NR	33.71 (3.12)	34.11 (3.54)	30.17 (2.98)	34.31 (3.59)	<.05
Abedi et al, 2010^16^	70.3 (12)	71.2 (14.4)	0.9	0.2	69.4	71.4	NS	30.1 (6.2)	30.8 (30.8)	29.7	30.9	NS
Al‐Mutairi et al., 2014^45^	99.3 (16.4)	98.6 (17.9)	12.96 (1.2)	−1.5 (0.5)	NR	NR	<.001	29.3 (4.2)	29.5 (5.2)	NR	NR	NR
Al‐Sarraj et al., 2009^17^	Group1:102.8 (24.2) Group 2: 91.4 (20)	NA	NR	NR	Group 1:94.2 (23.3) Group 2:86 (18.5)	NA	<.00001	Group 1:38.7 (7.6) Group 2:33.5 (6)	NA	Group 1:35 (6.6) Group 2:31.5 (5.8)	NA	<.0001
Azadbakht et al, 2005^24^	Group 1: Men: 86 IQR (79, 92) Women: 70 SD (11) Group 2: Men: 87 IQR (78, 95) Women: 71 SD (10)	Men: 84 IQR (78, 93) Women: 70 SD (12)	NR	NR	Group 1: Men: 73 IQR (68, 79) Women: 58 SD (7) Group 2: Men: 71 IQR (67, 78) Women: 57 SD (6)	Men: 84 IQR (75, 93) Women: 71 SD (12)	Intragroup: Group 1: Men: .03 Women: .04 Group 2: Men: .03 Women: .03 Group 1 vs control: <.05 Group 2 vs control: <.001	Group 1:29.9 (10.1) Group 2:29.8 (10.3)	29.5 (9.9)	NR	NR	NR
Azadbakht et al, 2007^23^	Group 1:70.1 (0.8) Group 2:70 (0.8) Group 3:70 (0.9)	NA	NR	NR	Group 1:70.4 (0.8) Group 2:70.7 (0.9) Group 3:70.1 (0.9)	NA	.57	NR	NR	NR	NR	NR
Darvish et al, 2012^46^	Group 1:72.1 (13.1)	71.9 (9.7)	NR	NR	Group 1:70.7 (11.6)	71.4 (9.2)	NS	Group 1:28.6 (5.8)	28.6 (3)	Group 1:28.1 (5.2)	28.4 (2.8)	NS
Delvarianzadeh et al., 2006 25	Group 1:73.1 (15.9)	74 (12)	NR	NR	Group 1:71 (14.6)	75.6 (11.2)	NS	NR	NR	NR	NR	NR
Elhayany et al., 2010^20^	Group 1:86.7 (14.3) Group 2: 85.5 (10.6) Group 3: 87.8 (13.7)	NA	NR	NR	Group 1:77.8 (13.1) Group 2: 78.1 (9.9) Group 3: 80.2 (13.2)	NA	Change over time *P* value: <.001; *P*‐value between diets: .557	Group 1:31.4 (2.8) Group 2:31.1 (2.8) Group 3: 31.8 (3.2)	NA	Group 1:28.1 (2.8) Group 2:28.5(2.9) Group 3: 29 (3.3)	NA	Change over time *P* value: <.001; *P*‐value between diets: .359
Esmaeili et al., 2014^47^	83.7 (14.89)	79.77 (12.65)	1.44	0.25	82.26 (15.67)	79.52 (12.7)	.004	33.32 (5.55)	32.61 (4.59)	32.73 (5.78)	32.51 (4.56)	.001
Fathi et al, 2016^48^	Group 1:74.6 (71.2, 78.1) Group 2:74.2 (71.8, 76.7)	75.1 (72.2, 78)	Group 1:2.1 CI (1.9,2.2) Group 2:2.2 CI (1.9,2.5)	1.2 CI (0.9,1.4)	NR	NR	Group 1 compared to control <.001 Group 2 compared to control <.001	Group 1: 28.8 CI (27.9, 29.6) Group 2:29.5 CI (28.7, 30.3)	28.9 CI (28.1, 29.7)	Change in BMI Group 1: −0.8 CI (−0.8, −0.7) Group 2: −0.9 CI (−1.0,‐0.8)	Change in BMI −0.4 CI (−0.6, −0.3)	Group 1 compared to control <.001 Group 2 compared to control <.001
Hariri et al., 2014^15^	70.84 (2.41)	71.61 (2.55)	NR	NR	70.4 (2.33)	71.21 (2.56)	.964	26.68 (0.71)	26.58 (0.73)	26.65 (0.68)	26.33 (0.74)	.309
Hosseinpour‐Niazi et al., 2015^49^	NR	NR	NR	NR	NR	NR	NR	Group 1:27.7 (0.6) Group 2:27.8 (0.6)	NA	Group 1:27.2 (0.6) Group 2:27.9 (0.6)	NA	NS
Jahangiry et al., 2016^50^	87 (16)	88 (14)	NR	NR	83 (15)	87 (12)	.046	29.8 (4.7)	30.5 (4.5)	28.6 (4.4)	29.5 (3.5)	.195
Kalter‐Leibovici et al., 2010^19^	Group 1:87.9 (9.6) Group 2:87.7 (8.3)	NA	Group 1:2.4 (5.9) Group 2: −0.4 (4.3)	NA	NR	NR	<.001	NR	NR	NR	NR	NR
Madjd et al., 2015^50^	Group 1:87.9 (9.9) Group 2:88.7 (8.9)	NA	NR	NA	Group1:80.3 (10.2) Group 2:79.9 (8.3)	NA	.015	Group 1:33.5 (3.6 Group 2:33.9 (3.0)	NA	Group 1:30.6 (3.8) Group 2:30.6 (2.8)	NA	.002
Madjd et al., 2016^52^	Group 1:82.45 (11.01) Group 2:82.69 (9.87)	NA	NR	NA	Group 1:77.42 (10.94) Group 2:77.39 (9.68)	NA	.248	Group 1:32.05 (3.94) Group 2:32.14 (3.20)	NA	Group 1:30.08 (3.86) Group 2:30.08 (3.15)	NA	.296
Mahdavi et al., 2016^53^	Group1: 85.37 (11.44) Group 2:84.72 (12.11)	NA	NR	NR	Group 1:75.50 (7.77) Group 2:72.90 (13.16)	NA	.011	Group 1:33.28 (2.90) Group 2:33.22 (3.16)	NA	Group 1:30.21 (2.03) Group 2:28.54 (3.28)	NA	.018
Mohammad‐Shahi et al., 2015^54^	NR	NR	NR	NR	NR	NR	NR	34.9 (3.9)	34.7 (5.07)	34.3 (4.4)	34.5 (5.11)	.845
Nourieh et al., 2012^22^	NR	NR	Percentage weight loss Group 1:1.62 (0.36) Group 2:1.75 (0.28)	NR	NR	NR	.79	NR	NR	NR	NR	NR
Parham et al., 2014^55^	NR	NR	NR	NR	NR	NR	NR	See BMI change	See BMI change	BMI change Group1: −0.76 (1.32)	BMI change −0.26 (1.19)	.08
Pourahmadi et al., 2015^21^	71.82 (1.31)	72.39 (1.19)	NR	NR	71.83 (1.32)	72.38 (1.19)	.75	28.22 (0.35)	28.28 (0.29)	28.23 (0.35)	28.29 (0.29)	.88
Rahimian et al., 2010^56^	Group 1:80.8 (12.1) Group 2:89.3 (12.4)	NA	NR	NA	Group 1:77 (12.1) Group 2:87.2 (11.8)	NA	.088	Group 1:32.2 (3.7) Group 2:36.4 (5.2)	NA	Group 1:30.7 (3.6) Group 2:35.5 (4.8)	NA	.028
Rajaie et al., 2012^57^	Group 1:80.9 (14.5) Group 2:79.7 (14.4)	NA	Group 1:1.70 (0.36) Group 2:1.72 (0.40)	NA	Group 1:79.2 (14.5) Group 2:78 (13.5)	NA	.96	Group 1:32.2 (5) Group 2:31.7 (5)	NA	Group 1:31.5 (5.1) Group 2:31.3 (4.8)	NA	.90
Ramezankhani et al., 2015^58^	Group 1: 76.32 (6.89) Group 2: 78.59 (7.54) Group 3: 78.6 (7.77)	76.48 (6.57)	NR	NR	Group 1: 71.92 (6.53) Group 2: 73.58 (6.75) Group 3: 73.67 (6.94)	77.17 (6.75)	Group 1: <.001 Group 2: <.001 Group 3: <.001 Control: .06	Group 1: 31.3 (2.44) Group 2: 31.5 (2.47) Group 3: 31.32 (2.51)	31.95 (2.44)	Group 1: 29.49 (2.18) Group 2: 29.49 (2.2) Group 3: 29.34 (2.20)	32.23 (2.48)	Group 1: <.001 Group 2: <.001 Group 3: <.001 Control: .051
Salar et al., 2016^59^	Group 1:78.39 (7.26) Group 2:75.24 (5.34)	79.66 (6.55)	Group 1:0.86 (1.68) Group 2: −0.36 (1.35)	−0.16 (1.49	NR	NR	.265	Group 1:29.81 (2.81) Group 2:29.37 (2.44)	30.54 (2.68)	NR	NR	NR
Shai et al, 2008^27^	Group 1:91.3 (12.3) Group 2:91.1 (13.6) Group 3:91.8 (14.3)	NA	Group 1:2.9 (4.2) Group 2:4.4 (6) Group 3: 4.7(6.5)	NA	NR	NA	Reduction greater in Groups 2 and 3 (<.001 vs .9 (Group 1)	NR	NR	NR	NR	NR
Tabesh et al., 2012^47^	Group 1:73.11 (2.03) Group 2:70.58 (2.3)	Group 3: 69.52 (2.76)	Group 1:3.62 (0.35) Group 2:3.36 (0.46)	Group 3: 3.44 (0.42)	NR	NR	.9	Group 1: 29.25 (0.88) Group 2: 29.83 (0.68)	Group 3: 29.2 (1.26)	NR	NR	.9
Razavi Zade et al., 2016^60^	Group 1:81 (8.9) Group 2:77.8 (10.1)	NA	Group 1:3.8 (2.2) Group 2:2.3 (1.7)	NA	Group 1:77.2 (7.9) Group 2:75.5 (9.3)	NA	Weight loss: .006	Group 1:28.5 (3.2) Group 2:28.3 (3.3)	NA	Group 1:27.2 (2.9) Group 2:27.5 (3)	NA	BMI change: .01

Abbreviations: NA, not available; NR, not reported; NS, not significant.

Outcomes reflecting glycaemic control were of secondary interest in this review. The most common reported glycaemic outcomes were fasting blood glucose (59%), insulin (31%), and HOMA‐IR (31%). Only four studies (14%) reported changes in HbA1c levels and two studies (7%) reported 2‐hour post prandial glucose levels. The average reduction in fasting blood glucose ranged from −10 to 99 mg/dL in the various interventions. The largest reductions in fasting blood glucose were seen in studies that limited CHO intake^20^, ^25^ and in intervention groups using Mediterranean, and ADA diets.^20^, ^27^ Shai et al.^27^ compared reduction in fasting blood glucose between diabetic and nondiabetic subjects in the various diets. Although there was no significant difference between the various diet groups in the nondiabetic participants, for the diabetic subjects the Mediterranean diet led to a significantly larger reduction (32.8 md/dL) compared to the low fat diet (−12.1 mg/dL) at 2 years (*P* < .001). Reduction in insulin levels ranged from −2.3 to 5.1 mU/mL across interventions with the largest reduction seen in a CHO‐restricted diet.^17^ The average reduction in HOMA‐IR varied from 0.1‐1.74 units.

### 3.4 Study quality and risk of bias assessment

There was a paucity of full descriptions for the weight loss interventions. Figure 2 provides a summary Template for Intervention Description and Replication for population health and policy interventions (TIDieR‐PHP) checklist for all the included studies. The majority of studies (79%) did not adequately describe the materials used in the intervention. Approximately half the studies did not report if the interventions were tailored to individuals and none reported any modifications to the intervention after study initiation. Most studies did not detail where the interventions were delivered (79%), the mode of delivery (62%) nor the duration and frequency of sessions (52%). Few studies (7%) described the intervention provider expertise and any relevant training they received for intervention delivery.

**FIGURE 3:**
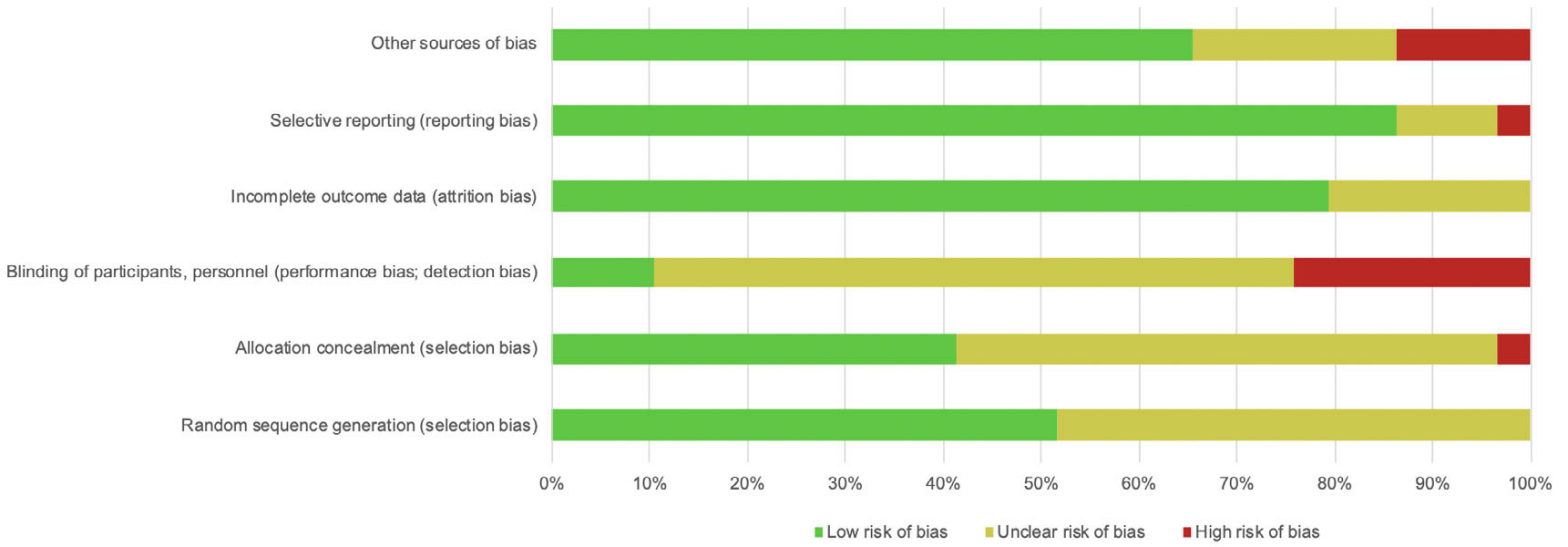
Summary of quality assessment for all studies

**FIGURE 4:**
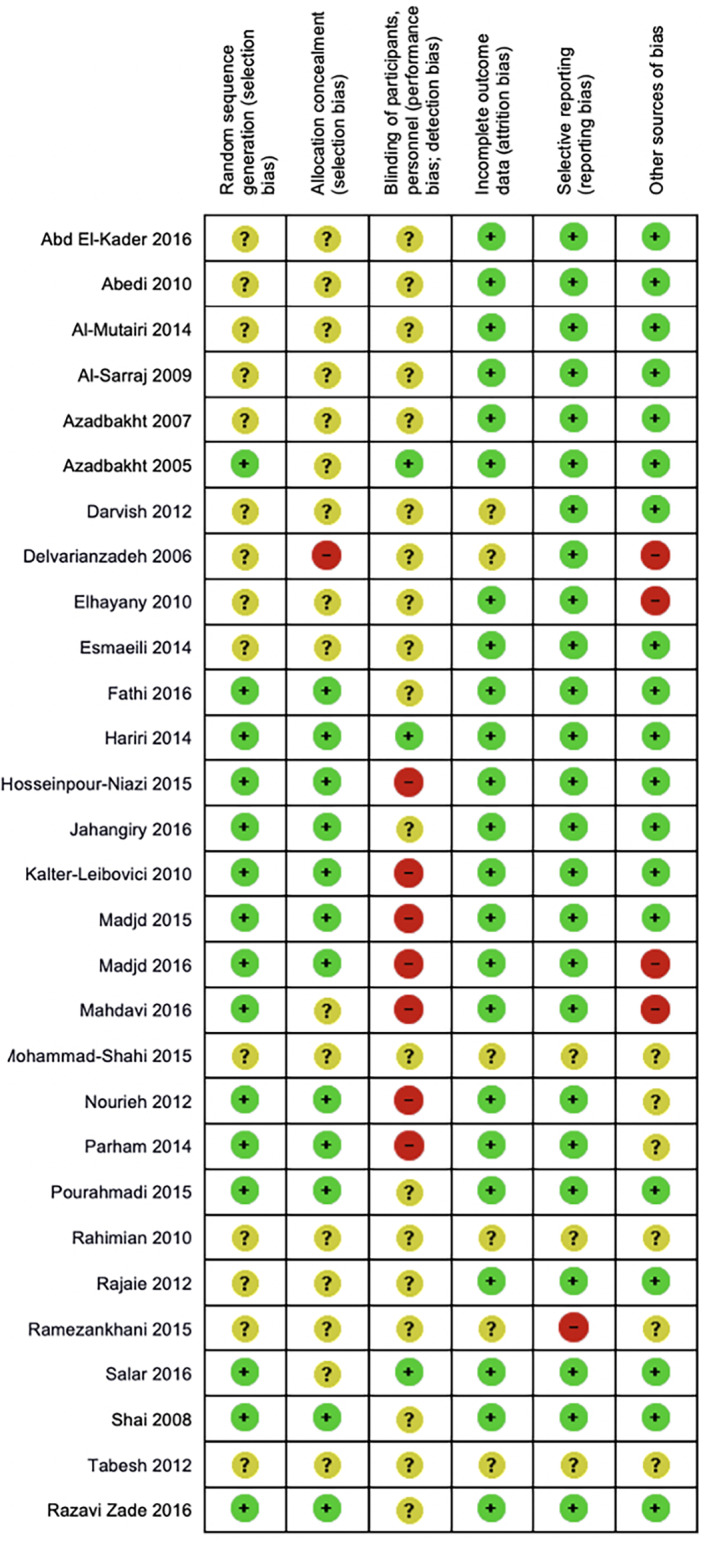
Quality assessment of individual studies

Figures 3 and 4 provide the risk of bias assessment for all the studies. Only 12 (41%) of RCTs reported on methods of randomisation and allocation concealment that were determined to be at low risk of bias. Masking of the participants and study personnel was rarely possible. These were the highest sources of bias collectively from all studies. The majority of studies were found to have low risk of bias for selective reporting and attrition (86% and 79%, respectively). About half the studies (48%) did not report on the methods used for random sequence generation appropriately and hence had an unclear selection risk of bias. Thirty‐four percent of studies has unclear or high risk for other sources of bias. Only one study^15^ reported low risk of bias for all items.

## 4 DISCUSSION

This review has for the first time systematically identified, summarized, and reviewed evidence from RCTs using dietary interventions for weight loss that have been conducted in the MENA region. The aim of this review was to provide benchmark data on the effectiveness and quality of weight loss interventions conducted in the MENA region. The studies examined included mainly middle‐aged females and weight loss observed ranged from −0.7 to 16 kg; the average weight loss was 4.8 kg. Importantly, the duration of most interventions was 3 months and given that obesity is a chronic disease, further evidence from longer term studies is required.

Understanding the limitations of the available evidence will help pave the way for future research that will inform obesity prevention and treatment guidelines for the region. Our review identified that many of the interventions were not described in sufficient detail with meaningful findings for implementation into healthcare. There were key deficiencies in description of the methodology that hinder replication and further validation. The focus of several studies was effects on various biomarkers rather than other clinical outcomes. The majority of studies had small sample sizes, with little information regarding the basis for the sample size enrolled. Most studies had an unclear or high risk of bias.

Most of the included studies (79%) were conducted in Iran. The remainder of studies were conducted in four other countries. Therefore, all interventions were conducted in five out of the total 22 selected countries from the MENA region. Considering the high prevalence of obesity in the Gulf Cooperation Council countries, few dietary weight loss trials have been conducted in these countries. Given the prevalence of obesity and NCDs in the MENA region, there is a need to conduct studies and have greater focus on obesity in the unrepresented countries. Furthermore, there have been no multicentre studies conducted in the region, and no study included more than one country or nationality of participants. In the future, there is a need for countries in the MENA region to collaborate and develop more generalizable evidence‐based approaches for the prevention and treatment of obesity.

Most RCTs recruited middle‐aged individuals. The rising prevalence of obesity in younger age groups^1^ in the MENA is alarming. Developing obesity at a young age can result in significant morbidity and mortality.^29^, ^30^, ^31^, ^32^ A study following a cohort of young men (n = 6502, age 22y) in Denmark, showed that obesity was associated with serious adverse outcomes. Forty‐eight percent of those with obesity had developed diabetes, CVD, venous thromboembolism, or had died before the age of 55 years.^33^ A disease simulation model using data from the US National Nutrition and Examination Survey reported that younger individuals with obesity lost more years of life: 0.8 years for men (60‐79 years old) vs 5.9 years for those aged 20‐39 years.^34^ For men with the highest BMI, the years of life lost for the older and younger group were 0.9 years vs 8.4 years, respectively. This evidence indicates that intervention in younger age groups may reduce morbidity and mortality significantly. The population in the MENA region is young with high obesity prevalence. In particular, obesity may be perpetuated through the intrauterine environment of young women with obesity, gestational diabetes, and T2DM. Examining the outcomes of weight loss interventions in this group is essential.

While several studies reported on weight loss and glycaemic status, none of the trials reported on diabetes remission as an outcome. T2DM remission is increasingly a realistic goal with evidence supporting that weight loss is an effective management for the prevention,^35^ improvement^36^ and remission^37^, ^38^ of T2DM. More studies reporting on changes in glycaemic status and diabetes remission are needed to reduce the burden of metabolic disease.

Combining exercise with dietary interventions has a positive impact on weight loss and its maintenance and also on obesity complications.^39^ A recent review examined physical activity interventions in Arabic speaking countries and found that the majority of the interventions (97%) resulted in an improvement of measured health outcomes (*P* < .05).^40^ Only four (13.7%) of the reviewed trials in our review integrated an exercise component in the intervention. The reported exercise interventions were not structured nor progressive and only aerobic exercise was recommended. Although our review does not include physical activity interventions without a dietary component, evidence shows that physical activity is beneficial. Considering the higher prevalence of obesity in women in the MENA region with considerably lower population levels of physical activity, future interventions should test the effectiveness of increasing physical activity levels in this population, particularly in the women.

Many of the trials reviewed recruited only women (55%), and only three trials (10%) had less than 50% women. Although obesity is more prevalent in women^1^ both worldwide and in countries in the MENA region,^41^ it is also important to test the effectiveness of dietary interventions in men who are also experiencing a rise in the prevalence of obesity and its complications.

The absence of direct expert clinical supervision and multi‐professional collaboration in the reported studies is notable. Also, multidisciplinary approaches with structured interventions combining dietary interventions with exercise and/or behavioural support were rarely used. The results of RCTs using multidisciplinary approaches and evidence based clinical expertise will provide key information about the applicability and acceptability of lifestyle interventions for weight loss in this region.^42^

Over the past few decades, the MENA region has witnessed major economic, social, lifestyle, and political changes that have potentially contributed to the rise in obesity prevalence. There are many genetic, geographical, cultural, and lifestyle patterns that set the MENA region apart from the rest of the world and that could affect the success of weight loss interventions. However, none of the included studies assessed quantitatively or qualitatively the factors that are associated with successful weight loss in this population. This could be a consideration for future studies in order to improve weight loss services in this region.

Our review was comprehensive in including studies irrespective of date of publication or language. The studies identified also included those with obesity complications and comorbidities. The studies, however, were too diverse to include in a meta‐analysis. Clinical research is developing in the MENA region and there is a greater acceptance of clinical research participation amongst the MENA population.^43^ It is envisaged that greater quality studies will emerge and increasing cooperation amongst MENA countries will result in a stronger evidence base for obesity to be tackled in the region.

## 5 CONCLUSION

Despite the rise in obesity prevalence in the MENA region, the RCTs examined in this review reported a wide range of weight loss responses to the interventions employed. Most interventions were adopted from interventions conducted in the western world, which may not be suitable for the MENA region. The short‐term duration of interventions is also problematic as obesity is chronic disease. Several deficiencies were noted in the reporting the methodological aspects of the studies and future studies should include full descriptions of the interventions, study design, and study conduct. Our review identified that culture sensitive studies with longer duration of follow‐up and evidence‐based designs are needed to adopt and deliver effective interventions for the treatment of obesity in the MENA region.

## CONFLICT OF INTEREST

No conflict of interest was declared.

## AUTHOR CONTRIBUTIONS

Shahrad Taheri planned the study. Shahrad Taheri, Hadeel Zaghloul and Odette Chagoury contributed to the design the study. Hadeel Zaghloul, Hadya Elshakh and Abdullah Elzafarany reviewed the publications. Hadeel Zaghloul, Shahrad Taheri, Barbara McGowan and Odette Chagoury produced the early drafts. All authors contributed to the final draft.

## Supporting information


**Appendix** **S1**: Supporting information.
