# Supplementary material for: A systematic review of randomized controlled trials of dietary interventions for weight loss in adults in the Middle East and north Africa region
Source: Clin Obes. 2020 Dec 26;11(2):e12434. doi: 10.1111/cob.12434 (PMC7988652; doi:10.1111/cob.12434)
Supplement: Supplementary file 1 — Appendix S1: Supporting information. [file COB-11-e12434-s001.docx]

Supplementary Table 1. Template for Intervention Description and Replication (TIDieR) checklist.


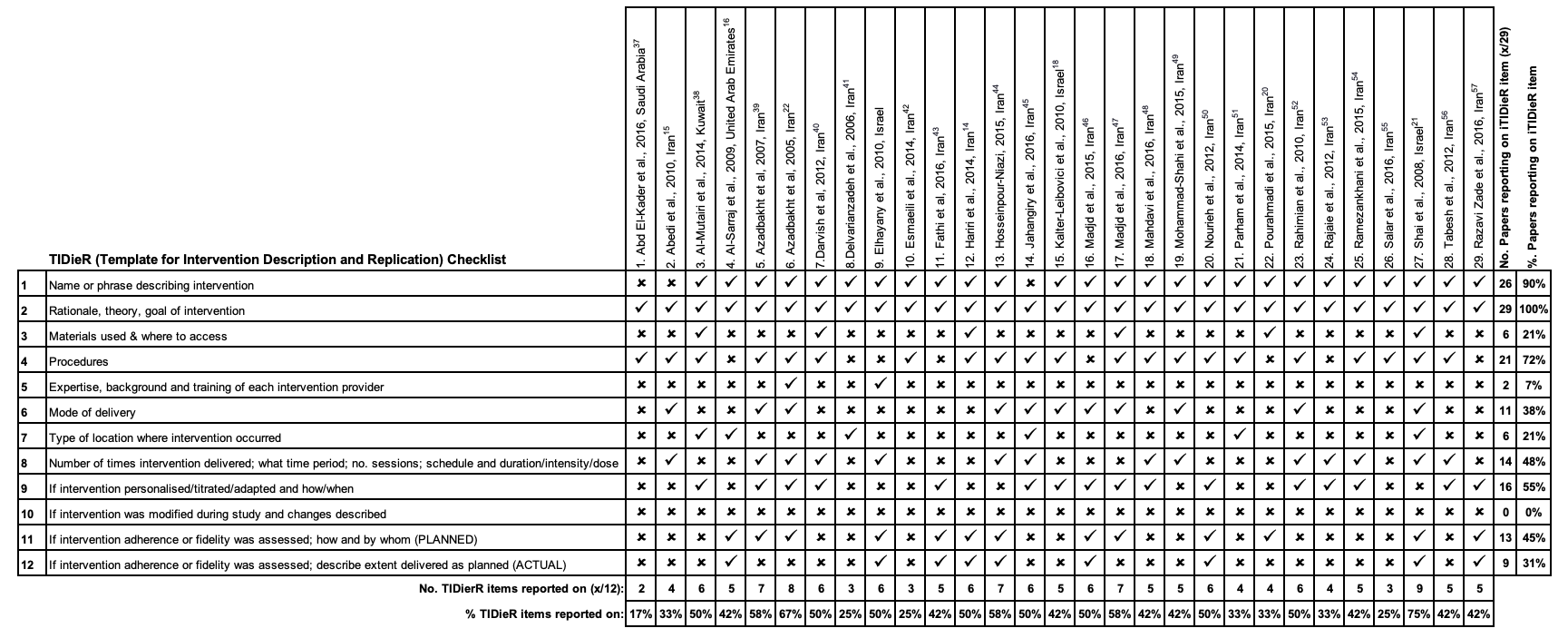


Supplementary Search Strategy:

((("Middle East and North Africa"[Title/Abstract] OR "MENA"[Title/Abstract] OR "middle east"[Title/Abstract] OR "middle eastern"[Title/Abstract] OR "middle easterner"[Title/Abstract] OR "middle easterners"[Title/Abstract] OR "near east"[Title/Abstract] OR "near eastern"[Title/Abstract] OR "near easterner"[Title/Abstract] OR "near easterners"[Title/Abstract] OR "Gulf Cooperation Council"[Title/Abstract] OR "GCC"[Title/Abstract] OR "Levant"[Title/Abstract] OR levantine*[Title/Abstract] OR "Turkey"[Title/Abstract] OR "Turkish"[Title/Abstract] OR "Turks"[Title/Abstract] OR "Persia"[Title/Abstract] OR Persia*[Title/Abstract] OR "Iran"[Title/Abstract] OR Iranian*[Title/Abstract] OR "Israel"[Title/Abstract] OR Israel*[Title/Abstract] OR "Saudi Arabia"[Title/Abstract] OR "Saudi Arabian"[Title/Abstract] OR "Saudi Arabians"[Title/Abstract] OR Saudi*[Title/Abstract] OR "KSA"[Title/Abstract] OR "kingdom of Saudi Arabia"[Title/Abstract] OR "United Arab Emirates"[Title/Abstract] OR "UAE"[Title/Abstract] OR emirat*[Title/Abstract] OR "Iraq"[Title/Abstract] OR Iraqi*[Title/Abstract] OR "Lebanon"[Title/Abstract] OR Lebanon*[Title/Abstract] OR lebanese*[Title/Abstract] OR "Yemen"[Title/Abstract] OR Yemen*[Title/Abstract] OR "Qatar"[Title/Abstract] OR Qatari*[Title/Abstract] OR "Jordan"[Title/Abstract] OR Jordanian*[Title/Abstract] OR "Kuwait"[Title/Abstract] OR Kuwaiti*[Title/Abstract] OR "Bahrain"[Title/Abstract] OR Bahraini*[Title/Abstract] OR "Oman"[Title/Abstract] OR Omani*[Title/Abstract] OR "Tunisia"[Title/Abstract] OR Tunisian*[Title/Abstract] OR "Cyprus"[Title/Abstract] OR Cypriot*[Title/Abstract] OR "Egypt"[Title/Abstract] OR Egyptian*[Title/Abstract] OR "Syria"[Title/Abstract] OR Syrian*[Title/Abstract] OR "Arab"[Title/Abstract] OR "Arabs"[Title/Abstract] OR "Palestine"[Title/Abstract] OR Palestinian*[Title/Abstract] OR "Arabian gulf"[Title/Abstract] OR "Persian gulf"[Title/Abstract] OR "Abu Dhabi"[Title/Abstract] OR "Dubai"[Title/Abstract] OR "Sharjah"[Title/Abstract] OR "Ajman"[Title/Abstract] OR "Umm Al Quwain"[Title/Abstract] OR "Ras Al Khaimah"[Title/Abstract] OR "Fujairah"[Title/Abstract] OR "gaza strip"[Title/Abstract] OR "Middle East"[Mesh:NoExp] OR "Iran"[Mesh] OR "Iraq"[Mesh] OR "Israel"[Mesh] OR "Jordan"[Mesh] OR "Kuwait"[Mesh] OR "Lebanon"[Mesh] OR "Oman"[Mesh] OR "Qatar"[Mesh] OR "Saudi Arabia"[Mesh] OR "Syria"[Mesh] OR "Turkey"[Mesh] OR "United Arab Emirates"[Mesh] OR "Yemen"[Mesh] OR "Bahrain"[Mesh] OR "Tunisia"[Mesh] OR "Cyprus"[Mesh] OR "Egypt"[Mesh] OR "Africa, Northern"[Mesh:NoExp] OR "Persia"[Mesh] OR "Arabs"[Mesh] OR "Arab World"[Mesh]))) AND (("macrobiotic diet"[Title/Abstract] OR "macrobiotic diets"[Title/Abstract] OR "pritikin diet"[Title/Abstract] OR "low carbohydrate diet"[Title/Abstract] OR "low carbohydrate diets"[Title/Abstract] OR "low-carbohydrate diet"[Title/Abstract] OR "low-carbohydrate diets"[Title/Abstract] OR "low carb diet"[Title/Abstract] OR "low carb diets"[Title/Abstract] OR "low-carb diet"[Title/Abstract] OR "low-carb diets"[Title/Abstract] OR "carbohydrate restriction"[Title/Abstract] OR "carbohydrate-restriction"[Title/Abstract] OR "carbohydrate restricted diet"[Title/Abstract] OR "carbohydrate-restricted diet"[Title/Abstract] OR "carbohydrate restricting diet"[Title/Abstract] OR "carbohydrate-restricting diet"[Title/Abstract] OR "restricted diet"[Title/Abstract] OR "restricted diets"[Title/Abstract] OR "restricted-diet"[Title/Abstract] OR "restricted-diets"[Title/Abstract] OR "sugar restricted diet"[Title/Abstract] OR "sugar restricted diets"[Title/Abstract] OR "sugar-restricted diet"[Title/Abstract] OR "sugar-restricted diets"[Title/Abstract] OR "sugar restriction"[Title/Abstract] OR "sugar-restriction"[Title/Abstract] OR "low energy diet" [Title/Abstract] OR "low energy diets" [Title/Abstract] OR "low-energy diet"[Title/Abstract] OR "low-energy diets"[Title/Abstract] OR "energy restriction"[Title/Abstract] OR "energy-restriction"[Title/Abstract] OR "energy restricted diet"[Title/Abstract] OR "energy-restricted diet"[Title/Abstract] OR "energy restricting diet"[Title/Abstract] OR "energy-restricting diet"[Title/Abstract] OR "keto"[Title/Abstract] OR "ketogenic diet"[Title/Abstract] OR "ketogenic diets"[Title/Abstract] OR "ketogenic-diet"[Title/Abstract] OR "ketogenic-diets"[Title/Abstract] OR "ketogenesis"[Title/Abstract] OR "ketotic-diet"[Title/Abstract] OR "ketotic-diets"[Title/Abstract] OR "ketosis"[Title/Abstract] OR "fat restricted diet"[Title/Abstract] OR "fat restricting diet"[Title/Abstract] OR "fat restricted diets"[Title/Abstract] OR "fat restricting diets"[Title/Abstract] OR "fat-restricted diet"[Title/Abstract] OR "fat-restricting diet"[Title/Abstract] OR "fat-restricted diets"[Title/Abstract] OR "fat-restricting diets"[Title/Abstract] OR "fat restriction"[Title/Abstract] OR "fat-restriction"[Title/Abstract] OR "low fat diet"[Title/Abstract] OR "low-fat diet"[Title/Abstract] OR "low fat diets"[Title/Abstract] OR "low-fat diets"[Title/Abstract] OR "fat-free diet"[Title/Abstract] OR "fat free diet"[Title/Abstract] OR "fat-free diets"[Title/Abstract] OR "fat free diets"[Title/Abstract] OR "protein restriction"[Title/Abstract] OR "protein-restriction"[Title/Abstract] OR "protein restricted diet"[Title/Abstract] OR "protein-restricted diet"[Title/Abstract] OR "protein restricted diets"[Title/Abstract] OR "protein-restricted diets"[Title/Abstract] OR "protein restricting diet"[Title/Abstract] OR "protein-restricting diet"[Title/Abstract] OR "protein restricting diets"[Title/Abstract] OR "protein-restricting diets"[Title/Abstract] OR "low protein diet"[Title/Abstract] OR "low-protein diet"[Title/Abstract] OR "low protein diets"[Title/Abstract] OR "low-protein diets"[Title/Abstract] OR "low glycemic diet"[Title/Abstract] OR "low-glycemic diet"[Title/Abstract] OR "low glycemic diets"[Title/Abstract] OR "low-glycemic diets"[Title/Abstract] OR "vegetarian diet"[Title/Abstract] OR "vegetarian diets"[Title/Abstract] OR "vegetarianism"[Title/Abstract] OR "vegetarians"[Title/Abstract] OR "vegetarian"[Title/Abstract] OR "vegan diet"[Title/Abstract] OR "vegan diets"[Title/Abstract] OR "veganism"[Title/Abstract] OR "vegan"[Title/Abstract] OR "vegans"[Title/Abstract] OR "caloric restriction"[Title/Abstract] OR "caloric-restriction"[Title/Abstract] OR "caloric restricted diet"[Title/Abstract] OR "calorie restricted diet"[Title/Abstract] OR "calorie restricted diets"[Title/Abstract] OR "caloric-restricted"[Title/Abstract] OR "calorie-restricted diet"[Title/Abstract] OR "calorie-restricted diets"[Title/Abstract] OR "calorie restricted"[Title/Abstract] OR "calorie-restricted"[Title/Abstract] OR "low calorie diet"[Title/Abstract] OR "low-calorie diet"[Title/Abstract] OR "low calorie diets"[Title/Abstract] OR "low-calorie diets"[Title/Abstract] OR "low cal diet"[Title/Abstract] OR "low-cal diet"[Title/Abstract] OR "low cal diets"[Title/Abstract] OR "low-cal diets"[Title/Abstract] OR "high fat diet"[Title/Abstract] OR "high fat diets"[Title/Abstract] OR "high-fat diet"[Title/Abstract] OR "high-fat diets"[Title/Abstract] OR "high protein diet"[Title/Abstract] OR "high protein diets"[Title/Abstract] OR "high-protein diet"[Title/Abstract] OR "atkins diet"[Title/Abstract] OR "zone diet"[Title/Abstract] OR "weight watchers diet"[Title/Abstract] OR "south beach diet"[Title/Abstract] OR "raw food diet"[Title/Abstract] OR "mediterranean diet"[Title/Abstract] OR "paleo diet"[Title/Abstract] OR "paleolithic diet"[Title/Abstract] OR "stone age diet"[Title/Abstract] OR "caveman diet"[Title/Abstract] OR "dash diet"[Title/Abstract] OR "Nutrition Therapy"[Mesh:NoExp] OR "Diet Therapy"[Mesh:NoExp] OR "Diet, Carbohydrate-Restricted"[Mesh:NoExp] OR "Diet, Diabetic"[Mesh:NoExp] OR "Diet, Fat-Restricted"[Mesh:NoExp] OR "Diet, Gluten-Free"[Mesh:NoExp] OR "Diet, Mediterranean"[Mesh:NoExp] OR "Diet, Paleolithic"[Mesh:NoExp] OR "Diet, Protein-Restricted"[Mesh:NoExp] OR "Diet, Reducing"[Mesh:NoExp] OR "Diet, Vegetarian"[Mesh] OR "Ketogenic Diet"[Mesh:NoExp] OR "Diet"[Mesh:NoExp] OR "Diet, Atherogenic"[Mesh:NoExp] OR "Diet, High-Fat"[Mesh:NoExp] OR "Diet, Western"[Mesh:NoExp] OR "Energy Intake"[Mesh] OR "Fasting"[Mesh:NoExp] OR "Portion Size"[Mesh:NoExp] OR "Serving Size"[Mesh:NoExp] OR "Diet, Macrobiotic"[Mesh:NoExp]))
